# Cherry Tree Close: Achieving Accreditation

**DOI:** 10.1192/bjo.2025.10509

**Published:** 2025-06-20

**Authors:** Zaim Mohdesham, Ahmed Rozza, Kopal Tandon

**Affiliations:** Derbyshire Healthcare NHS Foundation Trust, Derby, United Kingdom

## Abstract

**Aims:** Cherry Tree Close, a mental health rehabilitation and recovery unit based in Derby, England was assessed in October 2023 to compare current standards against the quality standards outlined in the Standards for Inpatient Mental Health Rehabilitation Services 4th Edition though did not fulfil the required criteria to achieve accreditation. Since then, the service has made further developments.

This is a service evaluation study to compare the current service delivery of Cherry Tree Close, against the quality standards set out in the Standards for Inpatient Mental Health Rehabilitation Services 4th Edition document to identify progress towards achieving accredited status.

**Methods:** The electronic patient records of service users admitted to the ward were reviewed between November and December 2024. Service users and members of the multidisciplinary team were interviewed. A visual inspection of the unit was carried out. Relevant standard operating procedures were reviewed.

To achieve accreditation, services are required to meet 100% of type 1, 80% of type 2 and 60% of type 3 standards.

**Results:** For type 1 standards, 95/109 standards (87.1%) were achieved in 2024 when compared with 93/109 (85.3%) in 2023.

For type 2 standards, 39/50 standards (78.0%) were achieved in 2024 when compared with 38/50 (76.0%) in 2023.

For type 3 standards, 8/14 standards (57.1%) were achieved in 2024 when compared with 5/14 (35.7%) in 2023.

The improvement relates to development of a local community enhanced rehabilitation service and expansion of the multidisciplinary professionals including recruitment of team psychologists, assistant psychologists, and resident doctor in training.

The remaining unmet standards relate to the infrastructure of the unit, care pathways, staff training, supervision, wellbeing, and service management.

**Conclusion:** Currently, Cherry Tree Close has not fulfilled the criteria to achieve accreditation status.

Further meetings have been arranged with the transformation team to work on further developing the service towards achieving accreditation.

